# From Editors’ Desk

**DOI:** 10.4103/0970-2113.71934

**Published:** 2010

**Authors:** Virendra Singh

**Affiliations:** *Chief Editor, Lung India E-mail: editor@lungindia.com*

Observation, idea, hypothesis and testing of hypothesis are the four basic steps of research. We all observe and based on observations many of us get an idea. Few brilliant people make a hypothesis based on such an idea. It is only a scientist who designs a proper study to test such a hypothesis and produce a piece of research. Research is fundamental need for scientific development of a society.

Treatment of respiratory ailments is important part of health care system. Though we have good international data on various respiratory problems yet simple extrapolation may fail to solve the problems related to Indian conditions. Therefore, we need to have Indian research data to draw appropriate inferences. During past few years respiratory physicians in India have collected good respiratory data.

Lung India is the premier journal publishing respiratory research in India. This status enjoyed by Lung India is because of the quality of research published. Fortunately, now, we are getting good quality research articles. In order to acknowledge the efforts of our research scientists contributing in Lung India, we are announcing awards in various categories. These will be annual awards and will be decided by an editorial committee, after publication of all the issues of that year. These awards will be given during the national conference of pulmonary diseases (NAPCON).

Your valuable feedback about the journal is food for our thought. Please do send your feeling about lung India regularly.

